# Bioengineering Liver Transplantation

**DOI:** 10.3390/bioengineering6040096

**Published:** 2019-10-16

**Authors:** Monique M.A. Verstegen, Bart Spee, Luc J.W. van der Laan

**Affiliations:** 1 Department of Surgery, Erasmus MC-University Medical Center Rotterdam, 3000 CA Rotterdam, The Netherlands; l.vanderlaan@erasmusmc.nl; 2 Department of Clinical Sciences of Companion Animals, Faculty of Veterinary Medicine, Utrecht University, 3584 CT Utrecht, The Netherlands; B.Spee@uu.nl

Since the first in-man liver transplantation was performed by Starzl et al. [1] in the early 1960s, many patients have successfully undergone organ transplantation. To date, over 30,000 liver transplantations are performed annually worldwide, which is estimated at less than 10% of the global need [2]. Transplantation is the only curative treatment for end-stage liver disease with different etiology. These include fat, alcohol- or viral hepatitis-related cirrhosis, liver cancer, inherited and metabolic diseases, and acute liver failure [3]. With demand increasing, alternatives for donor organs are urgently needed. In addition to optimizing donor/recipient selection procedures [4] and allocation systems to assure maximal utility of donor organs and equity to recipients [5], investing in innovating bioengineering technology remains important [6].

In the last decade, dynamic (hypo- or normothermic) machine perfusion protocols were introduced to preserve donor livers as opposed to static cold storage which, until then, was the standard [7,8,9]. In addition to preservation, normothermic machine perfusion can be used to assess the quality of the graft by monitoring liver function. Moreover, machine perfusion has the potential to improve grafts of marginal quality by applying treatment using (stem) cells, drugs, and compounds, for instance, to reduce steatosis, infection, or ischemia/reperfusion injury. Oxygenation of the liver graft seems to be a critical factor during machine perfusion. New developments are the use of gaseous oxygen, is based on venous systemic oxygen persufflation (OPAL) during static cold storage to improve hepatic energy homeostasis to prime the liver for the critical warm reperfusion which is known to be responsible for most of the ischemia/reperfusion injury [10]. As reported by Gallinat et al., this technique was shown to be safe and cost-effective, and demonstrated preliminary beneficial effects on clinical outcomes in a single-center randomized controlled clinical trial.

Potential alternatives for liver transplantation may include allogenic hepatocyte transplantation, in which the transplanted cells engraft the recipient’s liver and restore liver function [11]. So far, the success of hepatocyte transplantation depends on the disease type and level of cell replacement that is required to restore liver function. Although promising for some diseases, the long-term efficacy remains limited. Alternatively, genetics, gene-editing, and matrix-based culture methods of diseased hepatocytes could be employed to cure the patient’s own cells ex vivo as a personalized method for diseases with a known genetic mutation, as reviewed by Kruitwagen et al. [12]. Due to the (challenging) need of large numbers of hepatocytes coming from suboptimal livers that are unsuited for transplantation, cell function is often hampered [13]. To overcome this, (induced) pluripotent stem cells (iPSCs) or adult liver stem cells, such as cultured as liver organoids [14], might be a good source to use in cell transplantation [15]. Both cell types can be expanded to gain high numbers and could be initiated from the patient’s own tissue, preventing the need to treat the patient with lifelong immunosuppressive drugs. 

Next to functional cells, the actual organ scaffold is of key importance for the maintenance and potential engineering of functional liver tissue. If cells lack this spatiotemporal control of physical and biochemical cues, they cannot properly function and likely dedifferentiate [16]. A supporting scaffold that provides physical and biochemical characteristics, such as stiffness and matrix composition, is therefore essential [17]. Such scaffolds can be obtained from native liver tissue by decellularization [18,19,20]. These decellularized liver scaffolds can either be used for recellularization by infusion with cells [21] or processed into a biological hydrogels for clinical-grade expansion of stem cells or organoids [22]. Scaffolds can also be made from synthetical hydrogels, as reviewed by Ye et al. [23,24,25], each having their own (dis)advantages. Combining natural with synthetic hydrogels might increase the overall performance of tissue-engineered liver grafts [26]. Hepatocyte viability and proliferation might even be improved by adding even more components to the scaffold, such as special conduction polymers and gelatin, chitosan, and hyaluronan [27]. Kryou et al. reviewed novel opportunities for printing technology to create 3-dimensional (3D) scaffolds that can be used as a basis for functional liver tissue [28]. Printing technology to customize biliary stents are summarized by Boyer et al. [29]. By infusing these stents with collagen, mesenchymal stromal cells, and patient-derived cholangiocytes [29], personalized tissue engineering is nearing clinical application. 

The diverse contributions in this Special Issue on Bioengineering Liver Transplantation provides an overview of the novel and exciting opportunities in the field of liver regenerative medicine, biomaterials, and stem cell research that can be applied in future transplantations and personalized treatments of end-stage liver disease. 

## References

[1] Starzl T.E., Marchioro T.L., Vonkaulla K.N., Hermann G., Brittain R.S., Waddell W.R. (1963). Homotransplantation of the liver in humans. Surg Gynecol Obs..

[2] Who-ONT-Collaboration Global observatory on donation and transplantation (godt) data. http://www.transplant-observatory.org/data-charts-and-tables/.

[3] Fox A.N., Brown R.S. (2012). Is the patient a candidate for liver transplantation?. Clin. Liver Dis..

[4] Flores A., Asrani S.K. (2017). The donor risk index: A decade of experience. Liver Transplant..

[5] Tschuor C., Ferrarese A., Kümmerli C., Dutkowski P., Burra P., Clavien P.-A., Lendoire J., Imventarza O., Crawford M., Andraus W. (2019). Allocation of liver grafts worldwide is there a best system?. J. Hepatol..

[6] Bizzaro D., Russo F.P., Burra P. (2019). New perspectives in liver transplantation: From regeneration to bioengineering. Bioengineering.

[7] Quillin Iii R.C., Guarrera J.V. (2016). “In 10 years” of debate: Pro—machine perfusion for liver preservation will be universal. Liver Transplant..

[8] Halazun K.J., Quillin R.C., Rosenblatt R., Bongu A., Griesemer A.D., Kato T., Smith C., Michelassi F., Guarrera J.V., Samstein B. (2017). Expanding the margins: High volume utilization of marginal liver grafts among >2000 liver transplants at a single institution. Ann. Surg..

[9] Ravikumar R., Jassem W., Mergental H., Heaton N., Mirza D., Perera M.T.P.R., Quaglia A., Holroyd D., Vogel T., Coussios C.C. (2016). Liver transplantation after ex vivo normothermic machine preservation: A phase 1 (first-in-man) clinical trial. Am. J. Transplant..

[10] Gallinat A., Hoyer D.P., Sotiropoulos G., Treckmann J., Benkoe T., Belker J., Saner F., Paul A., Minor T. (2019). Oxygen persufflation in liver transplantation results of a randomized controlled trial. Bioengineering.

[11] Gramignoli R., Vosough M., Kannisto K., Srinivasan R.C., Strom S.C. (2015). Clinical hepatocyte transplantation: Practical limits and possible solutions. Eur. Surg. Res..

[12] Kruitwagen H.S., Fieten H., Penning L.C. (2019). Towards bioengineered liver stem cell transplantation studies in a preclinical dog model for inherited copper toxicosis. Bioengineering.

[13] Ibars E.P., Cortes M., Tolosa L., Gómez-Lechón M.J., López S., Castell J.V., Mir J. (2016). Hepatocyte transplantation program: Lessons learned and future strategies. World J. Gastroenterol..

[14] Huch M., Gehart H., van Boxtel R., Hamer K., Blokzijl F., Verstegen M.M.A., Ellis E., van Wenum M., Fuchs S.A., de Ligt J. (2015). Long-term culture of genome-stable bipotent stem cells from adult human liver. Cell.

[15] Hirabayashi M., Goto T., Hochi S. (2019). Pluripotent stem cell-derived organogenesis in the rat model system. Transgenic Res..

[16] Lee J.S., Shin J., Park H.-M., Kim Y.-G., Kim B.-G., Oh J.-W., Cho S.-W. (2014). Liver extracellular matrix providing dual functions of two-dimensional substrate coating and three-dimensional injectable hydrogel platform for liver tissue engineering. Biomacromolecules.

[17] Arriazu E., Ruiz de Galarreta M., Cubero F.J., Varela-Rey M., Pérez de Obanos M.P., Leung T.M., Lopategi A., Benedicto A., Abraham-Enachescu I., Nieto N. (2014). Extracellular matrix and liver disease. Antioxid Redox Signal.

[18] Verstegen M.M.A., Willemse J., van den Hoek S., Kremers G.J., Luider T.M., van Huizen N., Willemssen F.E., Metselaar H.J., IJzermans J.N.M., van der Laan L.J.W. (2017). Decellularization of whole human liver grafts using controlled perfusion for transplantable organ bioscaffolds. Stem Cells Develpment.

[19] Bühler N.E.M., Schulze-Osthoff K., Königsrainer A., Schenk M. (2015). Controlled processing of a full-sized porcine liver to a decellularized matrix in 24 h. J. Biosci. Bioeng..

[20] Wu Q., Bao J., Zhou Y.-J., Wang Y.-J., Du Z.-G., Shi Y.-J., Li L., Bu H. (2015). Optimizing perfusion-decellularization methods of porcine livers for clinical-scale whole-organ bioengineering. BioMed Res. Int..

[21] Willemse J., Lieshout R., van der Laan L.J.W., Verstegen M.M.A. (2017). From organoids to organs: Bioengineering liver grafts from hepatic stem cells and matrix. Best Pract. Res. Clin. Gastroenterol..

[22] Ijima H., Nakamura S., Bual R.P., Yoshida K. (2019). Liver-specific extracellular matrix hydrogel promotes liver-specific functions of hepatocytes in vitro and survival of transplanted hepatocytes in vivo. J. Biosci. Bioeng..

[23] Ye S., Boeter J.W.B., Penning L.C., Spee B., Schneeberger K. (2019). Hydrogels for liver tissue engineering. Bioengineering.

[24] Mattei G., Di Patria V., Tirella A., Alaimo A., Elia G., Corti A., Paolicchi A., Ahluwalia A. (2014). Mechanostructure and composition of highly reproducible decellularized liver matrices. Acta Biomater..

[25] Mattei G., Magliaro C., Pirone A., Ahluwalia A. (2018). Bioinspired liver scaffold design criteria. Organogenesis.

[26] Huang H., Oizumi S., Kojima N., Niino T., Sakai Y. (2007). Avidin–biotin binding-based cell seeding and perfusion culture of liver-derived cells in a porous scaffold with a three-dimensional interconnected flow-channel network. Biomaterials.

[27] Tahmasbi Rad A., Ali N., Kotturi H.S.R., Yazdimamaghani M., Smay J., Vashaee D., Tayebi L. (2014). Conducting scaffolds for liver tissue engineering. J. Biomed. Mater. Res. Part A.

[28] Kryou C., Leva V., Chatzipetrou M., Zergioti I. (2019). Digital bioprinting for organ (or liver) transplantation. Bioengineering.

[29] Boyer C.J., Boktor M., Samant H., White L.A., Wang Y., Ballard D.H., Huebert R.C., Woerner J.E., Ghali G.E., Alexander J.S. (2019). 3d printing for bio-synthetic biliary stents. Bioengineering.

